# Compensatory brain activity pattern is not present in older adults during the n-back task performance—Findings based on EEG frequency analysis

**DOI:** 10.3389/fpsyg.2024.1371035

**Published:** 2024-04-11

**Authors:** Ludmiła Zając-Lamparska, Emilia Zabielska-Mendyk, Dariusz Zapała, Paweł Augustynowicz

**Affiliations:** ^1^Department of General and Human Development Psychology, Faculty of Psychology, Kazimierz Wielki University, Bydgoszcz, Poland; ^2^Department of Experimental Psychology, Institute of Psychology, The John Paul II Catholic University of Lublin, Lublin, Poland

**Keywords:** compensatory brain activity, cognitive aging, alpha oscillations, theta oscillations, EEG, n-back, working memory, executive control

## Abstract

**Introduction:**

Cognitive ability is one of the most important enablers for successful aging. At the same time, cognitive decline is a well-documented phenomenon accompanying the aging process. Nevertheless, it is acknowledged that aging can also be related to positive processes that allow one to compensate for the decline. These processes include the compensatory brain activity of older adults primarily investigated using fMRI and PET. To strengthen the cognitive interpretation of compensatory brain activity in older adults, we searched for its indicators in brain activity measured by EEG.

**Methods:**

The study sample comprised 110 volunteers, including 50 older adults (60–75 years old) and 60 young adults (20–35 years old) who performed 1-back, 2-back, and 3-back tasks while recording the EEG signal. The study analyzed (1) the level of cognitive performance, including sensitivity index, the percentage of correct answers to the target, and the percentage of false alarm errors; (2) theta and alpha power for electrodes located in the frontal-midline (Fz, AF3, AF4, F3, F4, FC1, and FC2) and the centro-parietal (CP1, CP2, P3, P4, and Pz) areas.

**Results:**

Cognitive performance was worse in older adults than in young adults, which manifested in a significantly lower sensitivity index and a significantly higher false alarm error rate at all levels of the n-back task difficulty. Simultaneously, performance worsened with increasing task difficulty regardless of age. Significantly lower theta power in the older participants was observed at all difficulty levels, even at the lowest one, where compensatory activity was expected. At the same time, at this difficulty level, cognitive performance was worse in older adults than in young adults, which could reduce the chances of observing compensatory brain activity. The significant decrease in theta power observed in both age groups with rising task difficulty can reflect a declining capacity for efficient cognitive functioning under increasing demands rather than adapting to this increase. Moreover, in young adults, alpha power decreased to some extent with increasing cognitive demand, reflecting adaptation to them, while in older adults, no analogous pattern was observed.

**Discussion:**

In conclusion, based on the results of the current study, the presence of compensatory activity in older adults cannot be inferred.

## Introduction

Preserved cognitive ability is one of the essential conditions for successful aging and good quality of life in late adulthood (e.g., Castro-Lionard et al., 2011; Hartley et al., 2018; Yagi et al., 2020). Simultaneously, a well-documented phenomenon characteristic of aging is a cognitive decline (e.g., Salthouse, 2004; Harada et al., 2013). This decline occurs along with changes in brain volume and activity (e.g., Raz et al., 2005; Dennis and Cabeza, 2008; Grady, 2008; Hedden et al., 2016). The changes include, among others, the loss of neurons and their morphological changes, a decrease in the number of synaptic connections (Dickstein et al., 2007; Fjell and Walhovd, 2010), neurotransmission disruptions (Zanto and Gazzaley, 2019; Chamberlain et al., 2021), a decrease in the volume of the gray matter, which is particularly pronounced in the prefrontal and parietal cortex, hippocampus, caudate nucleus, cerebellum, and corpus callosum, and a deterioration of the white matter, primarily in the frontal regions (Raz et al., 2005; Dennis and Cabeza, 2008; Fjell and Walhovd, 2010; Ritchie et al., 2015; Leong et al., 2017; Zanto and Gazzaley, 2019). The activity of specific brain regions also changes with age. However, while there is ample evidence of differences between older and young adults in task-related brain activity, the pattern of these differences is inconsistent (Spreng et al., 2010; Eyler et al., 2011). Some neuroimaging studies indicate that older adults show reduced brain activity compared to young adults when performing the same cognitive tasks (Logan et al., 2002; Grady et al., 2006; Dennis and Cabeza, 2008; Campbell et al., 2012; Grady, 2012; Podell et al., 2012). On the other hand, increased brain activity in older adults has also been observed in numerous neuroimaging studies (Cabeza et al., 2004; Gutchess et al., 2005; Greenwood, 2007; Cabeza and Dennis, 2012; Grady, 2012; Jiang et al., 2017; Koen and Rugg, 2019; Yao and Hsieh, 2022). Moreover, even the same brain regions can show increased or decreased activity in older adults, depending on the cognitive domain involved, the difficulty of the task during which the activity was measured, and the accuracy of the task performance (Mattay et al., 2006; Schneider-Garces et al., 2010; Spreng et al., 2010; Grady, 2012; Zajac-Lamparska, 2020; McDonough et al., 2022).

In this context, it is acknowledged that aging can also be related to positive processes that allow one to compensate for the decline and adapt to it, both at the cognitive and neural levels (Reuter-Lorenz and Cappell, 2008; Goh and Park, 2009; Barulli and Stern, 2013; Morcom and Johnson, 2015; McDonough et al., 2022). These phenomena include compensatory brain activity in older adults, which can be considered a manifestation of spontaneous neurocognitive plasticity (Reuter-Lorenz and Cappell, 2008; Cabeza and Dennis, 2012; Zajac-Lamparska, 2020). Compensatory brain engagement, broadly defined, refers to increased or additional activity in a particular brain region in older adults that accompanies their performance on a task at the same level as young adults. The term can also be used when increased brain activity is positively correlated with performance in older adults but not in young persons. In general, when someone performs better on cognitive tasks, it is regarded as evidence for ongoing compensation (Mattay et al., 2006; Goh and Park, 2009; Park and Reuter-Lorenz, 2009; Cabeza and Dennis, 2012; Grady, 2012; Morcom and Johnson, 2015; Zajac-Lamparska, 2020). However, increased activity may also be a manifestation of inefficiency in the use of neuronal resources or a reduced selectivity in the brain's responses to the demands of the current task, known as dedifferentiation (Dennis and Cabeza, 2008; Grady, 2012; Jiang et al., 2017; McDonough et al., 2022; Yao and Hsieh, 2022).

One of the most common recurring patterns of increased (and potentially compensatory) brain activity in older adults is the overactivation of the frontal and, more specifically, prefrontal brain regions (Park and Reuter-Lorenz, 2009). The crucial role of the prefrontal region in the adaptation to the age-related decline in cognitive efficiency is postulated by the CRUNCH hypothesis—Compensation-Related Utilization of Neural Circuits Hypothesis (Reuter-Lorenz and Cappell, 2008). The hypothesis assumes that the decreased efficiency of cognitive processes resulting from aging makes it necessary to engage greater neuronal resources to achieve results similar to those obtained by young individuals. This engagement is manifested through increased activity of specific brain regions of older adults, primarily the prefrontal cortex (PFC). The explanations given by the authors of the CRUNCH hypothesis for the unique role of PFC refer to the cognitive functions that are associated with it, i.e., executive control, including attentional selection, maintenance, inhibition, rule switching, and context processing.

As increased executive control, in general, may be adaptively engaged to meet the challenges of the environment and cognitive tasks, and its involvement may also be a mechanism for adaptation to one's aging (Reuter-Lorenz and Cappell, 2008). This fact is somewhat paradoxical since, at the same time, it is the prefrontal cortex that shows marked age-related losses, accompanied by deterioration in executive control (Zanto and Gazzaley, 2019). Therefore, the authors assume that the compensatory involvement of prefrontal regions may occur at lower levels of difficulty of cognitive tasks when the performance of older adults is not considerably different from that observed in young persons. On the other hand, at higher cognitive demands, when older adults reach the resource ceiling (when task difficulty exceeds their resources), we are more likely to observe reduced activity in older adults, accompanied by a deterioration in their cognitive performance (Reuter-Lorenz and Cappell, 2008; Kang et al., 2022).

The results of neuroimaging studies support the assumptions of the CRUNCH hypothesis. In a review based on 80 articles describing the association of brain activity measured by fMRI or PET with cognitive ability, 70% of the studies identified a positive correlation between the level of activity of specific brain regions (including frontal, parietal, and temporal) and the level of task performance in older adults. At the same time, this association was not observed universally and was primarily related specifically to frontal cortex activity (Eyler et al., 2011). Moreover, a systematic review with meta-analysis based on 80 independent samples derived from studies on such cognitive domains as perception, memory, and executive functions found that, in general, older adults engaged the prefrontal regions more than young adults. In addition, older adults who performed cognitively at a level similar to young adults involved the left PFC, while poorer-performing older adults engaged the right PFC (Spreng et al., 2010).

Finally, in a relatively recent longitudinal study (Chen et al., 2022), the participants were divided into four age groups—young, middle-aged, young-old, and very old adults. The last three groups were additionally subgrouped into those who established successful cognitive aging vs. average aging, based on longitudinal changes in cognition. In brain regions associated with the task being performed (involving memory), successful agers showed activity similar to young individuals. In contrast, average agers showed reduced activity compared to young participants and successful agers. Moreover, in young-old successful agers, additional activity was identified in the left superior frontal cortex and right orbitofrontal cortex, which the authors interpret as a manifestation of functional compensation in successful aging. However, the study findings sometimes turned out to be inconsistent with the CRUNCH hypothesis. For example, Jamadar (2020) tested the CRUNCH hypothesis using a visual-spatial working memory (WM) paradigm. The results revealed that fMRI-measured brain activity in older and younger adults was opposite to that predicted based on the theoretical model. At low difficulty levels, there was a trend toward greater fMRI activity in younger than older adults in many regions, including the left and right inferior frontal cortex. At intermediate difficulty levels, most regions showed little or no difference between age groups. Finally, older adults showed greater activity at the highest difficulty level than younger adults in all regions. The author also conducted a systematic review, which led her to conclude that, although the CRUNCH hypothesis is highly influential in scientific literature, a surprisingly small number of published studies directly and explicitly tested the predictions of this model (Jamadar, 2020).

What is essential is that fMRI and PET are the two most dominant neuroimaging methods in research on compensatory brain activity. The robustness of interpreting their results regarding cognitive processes could be substantially increased using other indicators, which would confirm the compensatory engagement of specific cognitive processes.

Increased cognitive control, top-down processing, and executive function engagement are reflected in the increase of theta rhythm (4–8 Hz) (Sauseng et al., 2010; Roux and Uhlhaas, 2014). Furthermore, researchers postulate that prolonged theta increase reflects a continuous process of cognitive processing—maintaining and manipulating information (McEvoy et al., 2001), corresponding to the top-down control and executive processes. Among the executive processes, WM engagement has also been systematically found to be related to a theta power increase (Klimesch, 1999; Roux and Uhlhaas, 2014). Moreover, theta power increases proportionally to increase WM processing load and successfully store larger stimuli sets (Belham et al., 2013; Itthipuripat et al., 2013; Constantinidis and Klingberg, 2016). Theta rhythm is supposed to be specifically engaged in the temporal organization of the elements maintained in WM and to be the basis of their ordinal arrangement (Hsieh et al., 2011; Roux and Uhlhaas, 2014). Additionally, it is essential to note that a theta synchronization increase and a further increase of this signal power with rising WM load is observed mainly for cortical regions: frontal, central, and parietal (McEvoy et al., 2001; Jensen and Tesche, 2002; Constantinidis and Klingberg, 2016).

In the literature, particular importance is attributed to frontal midline theta (FMT). This term was introduced by Ishihara and Yoshi (1972). There is general agreement that FMT is in the range of 4–8 Hz rhythmic activities and is usually maximal around the Fz electrode (Hsieh and Ranganath, 2014). There is a relationship between FMT oscillations and WM (Gevins et al., 1997; Scheeringa et al., 2009; Hsieh et al., 2011). Gevins et al. (1997) found that the direct FMT power increase accompanies the increase of WM load. Some findings also suggest that theta oscillations may be linked to context representation (Staudigl and Hanslmayr, 2013), as well as proactive control, response initiation, and response inhibition (Cooper et al., 2019; Messel et al., 2021). In general, FMT is conceptualized as a marker of the need for cognitive control (Cavanagh and Frank, 2014; Eschmann et al., 2018; Eisma et al., 2021).

Another neural phenomenon that can be linked to an increased engagement of control processes, primarily attentional, is a decrease in the alpha rhythm power (8–12 Hz). However, the functional interpretation of this rhythm is less unambiguous (Roux and Uhlhaas, 2014). This rhythm is associated mainly with active inhibition of information unrelated to the performed task, evidenced by increased alpha power in brain regions beyond those crucial for the task. It is observed also in the case of WM (Tuladhar et al., 2007; Haegens et al., 2010; Bonnefond and Jensen, 2012). On the other hand, the decrease in alpha power is interpreted as an increased engagement of the region in which this rhythm is observed during cognitive task execution or as a reinforcement of information flow efficiency in that region (Krause et al., 2000; Ward, 2003; Pesonen et al., 2007; Gajewski and Falkenstein, 2014).

In summary, an increase in theta power and a decrease in alpha power of a specific brain region are associated with the same phenomena, i.e., increased information flow and engagement in cognitive activity related to that region. As Gratton (2018) suggests, both theta and alpha rhythms are related to cognitive control. However, in a complementary way, i.e., alpha is related to proactive control (maintaining representations), while theta is related to reactive control (changes in representations). When attention needs to be directed toward incoming information, theta power increases, while alpha power becomes suppressed (Clements et al., 2021). From this perspective, it is essential to include the dynamics of theta and alpha rhythms in the study simultaneously.

The results of some studies on oscillations in old age can be interpreted in light of the models of compensatory brain engagement in late adulthood and the CRUNCH hypothesis, even when the authors themselves do not write about it. For instance, researchers observed that among individuals in early and middle adulthood, the theta increase in the frontal and central brain areas was linked to increasing difficulty of the WM task. In contrast, in older adults, the opposite was true—a lower WM load, which did not differentiate between younger and older adults in terms of task accuracy, was related to a theta level that was higher than that in the case of a higher memory load (McEvoy et al., 2001). It suggested an increased (possibly compensatory) engagement of the fronto-central network during lower cognitive demands and its impairment during higher demands. Moreover, for increasing task difficulty, young individuals exhibited a decreased alpha power in the parietal region but not the frontal one, while in older adults, the decrease was also evident in the frontal region. The authors interpret these findings as suggesting that older adults rely more on strategies based on frontal area engagement, which is in line with the assumptions of the CRUNCH hypothesis.

In another study, older participants manifested worse visual WM task performance and decreased theta and alpha power in frontal and central areas compared to young adults (Belham et al., 2013). A lower theta power can be interpreted as an indicator of age-related decline. A simultaneous alpha power decrease may also suggest additional neural engagement of the same regions, although in a task-unspecific (and hence less effective) way. In this case, only the alpha findings, but not the theta ones, corresponded to the CRUNCH hypothesis. Finally, studies including old and young adults and involving the n-back task at two levels of difficulty (0- and 2-back) showed that older adults, in comparison to younger participants, were characterized by lower performance in the 2-back condition, but not in the 0-back condition, and consistently manifested lower power of the frontal theta and parietal alpha as well as a strongly reduced alpha power in the frontal regions in the case of the more difficult 2-back condition (Gajewski and Falkenstein, 2014). According to the study's authors, the lower power of frontal theta may reflect a limited ability of older adults to engage frontal resources. At the same time, the authors link the decrease in alpha to the necessity to engage increased attentional resources for better task performance. These results can be interpreted as reflecting compensatory engagement, which is enough for the easy task but, at the same time, insufficient for increased cognitive demands, as postulated in the CRUNCH hypothesis.

In the context of the above considerations, the current study aimed to search for the electrophysiological (measured with EEG) indicators of compensatory brain activity in older adults. The reason is that the EEG-based approach requires hypotheses based not only on the localization of compensatory brain activity but also on the cognitive processes theoretically linked to the activity of interest. Since fMRI and PET findings cannot be directly expressed in EEG terms, the following reasoning has been employed to predict the results: compensatory brain overactivation observed in fMRI and PET (*prefrontal cortex*) → cognitive interpretation of the overactivation (*increased executive control*) → determination of the EEG indicators of the cognitive processes specified based on the interpretation (*increase in theta power, including FMT, and decrease in alpha power*) → the verification of the existence of the indicators in EEG signal. Accordingly, using EEG measurements, it might be possible to verify the compensatory role of specific processes beyond just monitoring the increased activity of particular brain areas.

In the study, we also decided to consider the task difficulty and the level of task performance of older and young adults, in line with the predictions made by the authors of the CRUNCH hypothesis presented above. Consequently, we intended to test the power of theta and alpha oscillations and the performance of the n-back task, depending on the level of the task difficulty, the age of the participants, and the interaction between these two variables.

**Figure 1:**
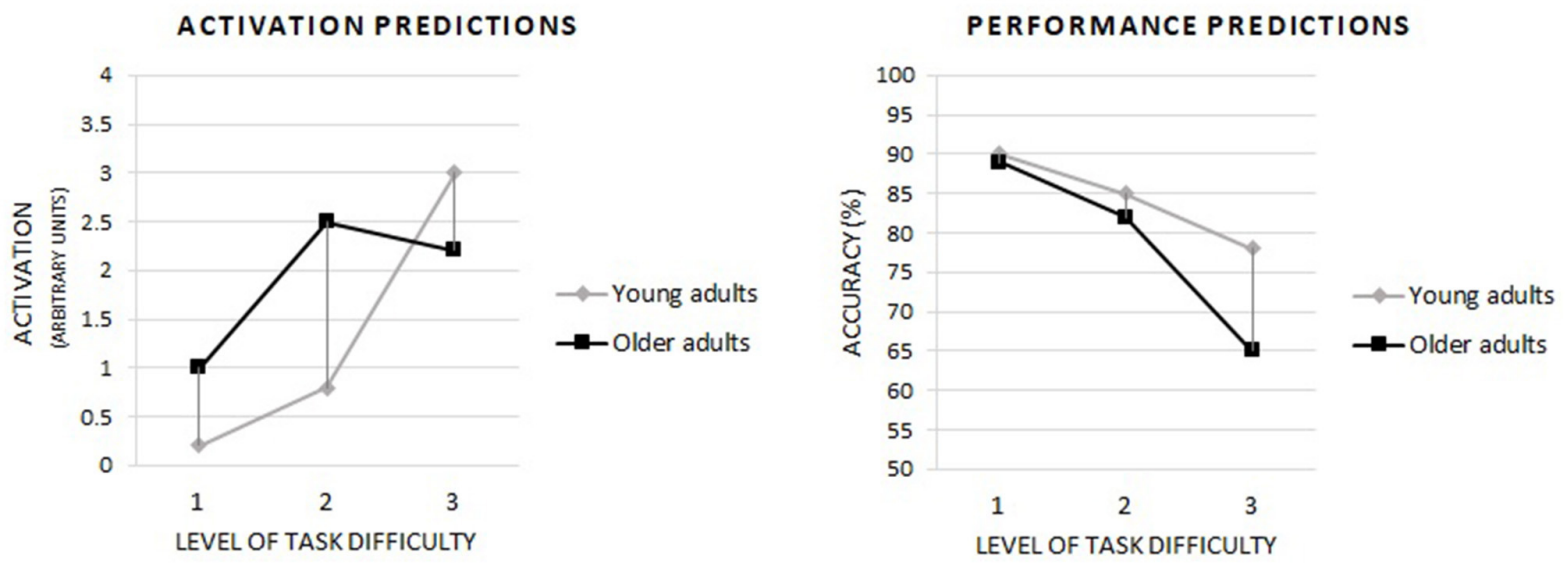
Based on the CRUNCH hypothesis, predictions of the occurrence of compensatory brain activity in older adults depending on the level of task difficulty and including the level of cognitive performance. Own drawing based on: Reuter-Lorenz and Cappell (2008), p. 180.

We expect that older adults will perform a task with the lowest difficulty level with the same accuracy as young adults. Further, we suppose that, as the task's difficulty level increases, the performance accuracy will decrease in both age groups. At the same time, the decrease in accuracy will be greater in older adults than in young adults. Consequently, in the more difficult versions of the task, the accuracy of older adults will be lower than that of young adults, and the age-related differences will be most remarkable in the most challenging version of the task. Our predictions for cognitive performance align with what is presented in Figure 1 (plot on the left), illustrating the assumptions of the CRUNCH hypothesis. Moreover, we hypothesize that at those levels of task difficulty at which the accuracy of older adults will be similar to young adults, theta power in the frontal and parietal regions will be higher in older adults than in young adults as an indication of compensatory involvement of executive control, according to the CRUNCH hypothesis (Figure 1, plot on the right). At the same time, we expect that theta power in older adults will only increase to a level of difficulty at which compensatory activity can maintain the cognitive performance of older adults at a level similar to young adults; at a level of difficulty too high to maintain such accuracy, the theta power will decrease, which is illustrated by Figure 1 (plot on the right). The pattern we expect regarding alpha power is the opposite of the theta power hypotheses discussed above. On the contrary, in young adults, we expected a steady increase in the theta power and a decrease in the alpha power with increasing cognitive demands as an expression of the adaptation to them.

## Methods

### Participants

The initial sample size was 117 individuals (54 older and 63 young adults). After excluding the sample participants whose EEG recordings were of insufficient quality, the final study sample comprised 110 participants, including 50 older and 60 young adults. The sociodemographic characteristics of participants are provided in Table 1.

**Table 1:** Sociodemographic characteristics of participants.

**Characteristic**	**Older adults (*n* = 50)**	**Young adults (*n* = 60)**	
Age: *M* (SD)	66.200 (3.774)	25.733 (5.155)	
**Gender**			
Women: *n* (%)	41 (82%)	40 (66.667%)	χ^2^ = 3.302 *p* = 0.069
Men: *n* (%)	9 (18%)	20 (33.333%)	
SPM raw score: *M* (SD)	42.900 (9.259)	52.733 (6.011)	*t* = −6.707 *p* < 0.001
MMSE: *M* (SD)	28.720 (1.051)	NA	
Years of education: *M* (SD)	14.790 (3.099)	16.017 (2.406)	*t* = −2.336 *p* = 0.021
**Level of education** ^a^			
Higher education: *n* (%)	21 (42%)	28 (46.667%)	χ^2^ = 7.521 *p* = 0.023
Secondary education/vocational secondary education: *n* (%)	21 (42%)	31 (51.667%)	
Basic vocational education: *n* (%)	8 (16%)	1 (1.667%)	
**Employment**			
Pensioner: *n* (%)	45 (90%)		
Employed: *n* (%)	5 (10%)	22 (36.667%)	
Own business: *n* (%)		4 (6.667%)	
Student: *n* (%)		30 (50%)	
Unemployed^b^: *n* (%)		4 (6.667%)	
**Type of work activity** ^c^			
Intellectual: *n* (%)	14 (28%)	6 (23.08%)	χ^2^ = 0.214 *p* = 0.859
Physical: *n* (%)	36 (72%)	20 (76.92%)	
Physical activity^d^: *n* (%)	30 (60%)	35 (58.333%)	χ^2^ = 0.313 *p* = 0.644
Intellectual activity^d^: *n* (%)	34 (68%)	27 (45%)	χ^2^ = 5.840 *p* = 0.016

Where appropriate, it was calculated whether the differences between the age groups were statistically significant.

^a^Levels of education by the Polish education system.

^b^Persons who are not employed and at the same time are not students or pensioners.

^c^For older adults, it refers to the previous or current work activity (n = 50); for young adults, it refers only to individuals who are employed and self-employed (n = 26).

^d^Physical/intellectual activity undertaken voluntarily, that is, not resulting from work and/or formal education.

Participants were volunteers who registered in response to advertisements distributed online and on public transport in Bydgoszcz (Poland). At the same time, participants had to meet the inclusion criteria, which were verified during the introductory meeting and which were as follows: (1) age: belonging to one of the age groups—older adults (60–75 years old) or young adults (20–35 years old). The 60–75 age group limits the influence of the advance of various age-related changes, and at the same time, this group is classified as early late adulthood. The 20–35 age group, on the other hand, falls within early adulthood (Sugarman, 2001); (2) no mental illness and neurological disorders, including neurodegenerative diseases or severe head injuries (verified by structured interview and the Mini International Neuropsychiatric Interview—M.I.N.I. 7.0); (3) normal or corrected-to-normal vision; (4) intellectual norm (verified with the Raven's Standard Progressive Matrices, SPM, in Polish standardization, which was performed without a time limit); (5) no dementia symptoms (verified in older adults with the polish version of the Mini-Mental State Examination, MMSE); (6) no visible abnormal brain activity in EEG (verifiable only in the pre-test); (7) signing an informed consent to participate in the study (after familiarizing oneself with the aim of the study and the conditions of participation, as well as having received satisfactory answers to all questions). The MMSE results for the older adults group and SPM results for both age groups are included in Table 1.

### Procedure and materials

The study protocol and the informed consent form were approved by the Bioethics Committee of the Nicolaus Copernicus University in Toruń at the Ludwik Rydygier Collegium Medicum in Bydgoszcz (KB 90/2018). Before enrollment in the study, each participant was informed about its purpose, anonymity, and the possibility of withdrawal from the study. All subjects gave written informed consent following the Declaration of Helsinki. The results presented here were collected in an initial measurement in a research project on the effects of cognitive training in older and young adults (National Science Centre, Poland, grant no 2017/25/B/HS6/00360). The study was registered after completion with a ClinicalTrials.gov Identifier: NCT06235840 on 31 January 2024.

The research was performed with each participant individually. The EEG signal was recorded while the study participants performed the n-back task at three levels of difficulty: 1-back, 2-back, and 3-back.

The n-back task is a well-known measure of WM efficiency in content updating (Jaeggi et al., 2010; Schmiedek et al., 2014). It measures two aspects of WM: capacity and executive control (Necka and Lulewicz, 2016). Moreover, research using event-related potentials (Chen et al., 2008) supported a model that indicates matching, replacement, and shift as three sub-processes involved in the n-back task, starting from the 2-back level (for 1-back, only matching and replacement are necessary). The performance of the n-back task is associated with the involvement of the frontal and parietal cortical regions (Owen et al., 2005). At the same time, the decline in working memory and executive control (including the n-back task performance) with age is a well-documented phenomenon (e.g., Braver and West, 2008; Nyberg et al., 2009; Harada et al., 2013; Schmiedek et al., 2014).

**Figure 2:**
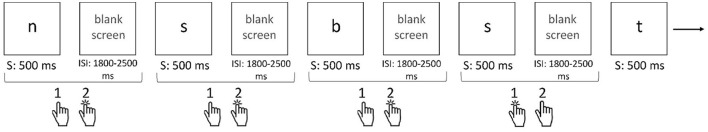
Single n-back task paradigm (example: 2-back). Description of 2-back task: On the computer screen, the letters appear and disappear one by one. The task is to decide whether the currently presented letter has appeared two items back. If yes, the respondent should press the “1” button on the response pad; if not, they should press the “2” button. For the sequence of letters shown in the illustration: n, **s**, b, **s**, t, the following sequence of reactions (button selections) is correct: 2, 2, 2, 1, and 2. S, stimulus presentation; ISI, inter-stimulus interval. Source: Zając-Lamparska (2021), p. 285.

The n-back task involves continuously presenting items (letters in this study) that appear and disappear one by one. During each presentation, the participant must judge whether the currently displayed item matches the item presented “*n*” trials earlier. Hence, the number “*n*” in the n-back task indicates with which item (presented how many trials before) the currently presented item should be matched (Zając-Lamparska, 2021, see Figure 2).

The n-back task used in the current study was programmed in PsychoPy software (Peirce, 2009). The order in which each task level was performed was fixed—always starting with the easiest (1-back) and ending with the most difficult (3-back) level. Each difficulty level comprised six blocks with 20+*n* (where *n* is the number from the n-back term) stimuli presented in each block. Among the stimuli in each block, seven were targets (i.e., items the same as those presented “*n*” trials back), with a total stimulus number of 21, 22, and 23 for 1-, 2- and 3-back tasks, respectively. The stimuli, including targets and non-targets, were presented randomly, i.e., the software randomized the order of stimulus presentation each time the task was run, respecting the rule on the number of targets and non-targets set for each level of the n-back task. The duration of the stimulus presentation was 500 ms. The inter-stimulus interval (ISI) was randomized from 1,800 to 2,500 ms. At the same time, the first block at each difficulty level was treated as a training block, i.e., familiarizing the participants with the rules of performing a task at a given difficulty level. The results obtained in these first blocks were not included in the statistical analyses. The instructions to participants focused on explaining how to answer correctly and did not include information to try to respond as quickly as possible.

The main indicator of the n-back task accuracy at each level of difficulty was the sensitivity index (*d*′), as a measure of discriminability based on signal detection theory. The sensitivity index (*d*′) was computed with the formula for yes/no tasks: *d*′ = *z*(*H*) – *z*(FA), where *H* and FA are the Hits and False Alarm rates and *z*(*H*) and *z*(FA) are the *z*-transformations, respectively (Stanislaw and Todorov, 1999). We calculated *d*′ in Microsoft Excel software using the formula: *d*′ = NORMINV (hit-rate, 0, 1)—NORMINV (false-alarm-rate, 0, 1). To adjust the extreme values, before the *z*-transformation, we substituted rates of 0 with “0.5/*n*” and rates of 1 with “(*n*-0.5)/*n*,” where “*n*” denotes the total number of signal or noise trials, respectively (Macmillan and Kaplan, 1985). In addition, the percentage of correct responses to the target and the percentage of false alarm errors, i.e., responses to non-targets that should have been inhibited, were taken into account.

### EEG data acquisition and processing

The EEG signal was recorded with a 32-channel electroencephalograph (BioSemi the Active Two System, sampling rate 2,048 Hz) using Ag/AgCl pin-type active-electrodes (left: Fp1, AF3, F3, F7, FC1, FC5, C3, CP1, CP5, T7, P3, P7, PO3, and O1; right: Fp2, AF4, F4, F8, FC2, FC6, C4, CP2, CP6, T8, P4, P8, PO4, and O2; midline: Fz, Cz, Pz, and Oz) arranged according to the international 10–20 system. Two electrodes, i.e., the Common Mode Sense (CMS) active electrode and Driven Right Leg (DRL) passive electrode, were used as a grounding system. The reference was the Cz electrode. The EEG signal was analyzed for correct responses to the target.

The EEG data were preprocessed by filtering out signals below 0.1 Hz and above 40 Hz using a finite impulse response filter. The data were then re-referenced using a common average reference. To remove short-time high-amplitude artifacts, the Artifact Subspace Reconstruction (ASR) method was used (as implemented in the Clean Raw Data EEGLab plug-in; Plechawska-Wojcik et al., 2019). Next, a series of techniques, including independent component analysis (ICA) decomposition “runnica” dipolar source estimation (DIPFIT) with a boundary element head model (BEM), and multi-variate source classification, were applied to identify the sources of brain oscillations based on an equivalent current dipole model (as implemented in ICLabel EEGLab plug-in; Pion-Tonachini et al., 2019). Independent components were classified based on their power spectrum distribution, time-frequency characteristics, and dipole localization, and all non-brain sources, such as eye blinking, lateral eye movement, muscle activity, and electrical noise, were removed. The remaining signal was segmented into segments, each lasting 1,200 ms, with a baseline from −200 to 0 ms and an event period from 0 to 1,000 ms. The segments were averaged with reference to individual subjects and experimental conditions (level of difficulty of the n-back task, age group) and then subject to a fast Fourier transform (FFT); a power spectrum (meaning the square root of the sum of the squared the FFT results) was calculated, and then, a decimal logarithm was computed. The procedure was applied to two frequency ranges: theta (4–7 Hz) and alpha (8–12 Hz).

### Statistical analyses

A mixed model two-way analysis of variance (ANOVA) with two factors: age group (young or old adults; between-subjects factor) and the difficulty level of the n-back task (three levels; within-subjects factor) was used to test the predictions made for both behavioral and electroencephalographic parameters. The parameters analyzed as dependent variables were (a) for behavioral data, the sensitivity index (*d*′), the percentage of correct responses to the target, and the percentage of false alarm errors for the three levels of difficulty of the n-back task; and (b) for EEG data, with theta and alpha power for electrodes located in the fronto-midline (Fz, AF3, AF4, F3, F4, FC1, and FC2) and centro-parietal (CP1, CP2, P3, P4, and Pz) areas, ANOVA was supplemented with *post-hoc* analysis with the use of the Tukey test for unequal sample sizes.

## Results

### Cognitive performance

In line with the hypotheses, the cognitive performance analyses concentrated on the accuracy of the n-back task performance. Additional analyses regarding response times can be found in the Supplementary material.

**Figure 3:**
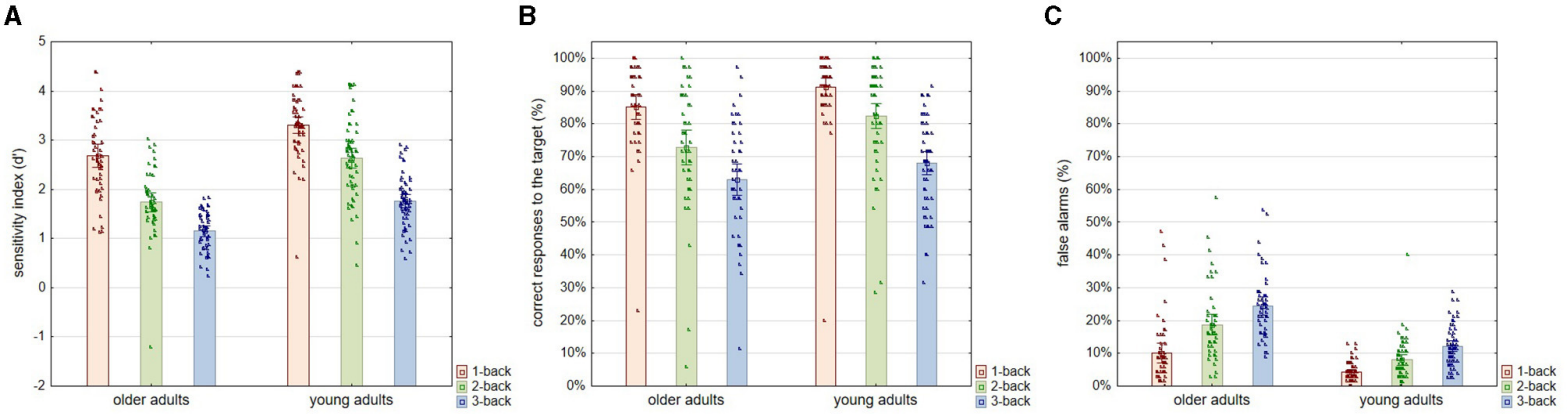
Sensitivity index (*d*′), correct answers to the target (%), and false alarm errors (%) for three levels of n-back task difficulty in older and young adult age groups. All plots provide the indicators of the n-back task performance according to the level of task difficulty in two age groups: older and young adults. Plot **(A)** shows the accuracy in the n-back task indicated by the sensitivity index (*d*′). Plot **(B)** shows the percentage of correct responses to the target stimuli. Plot **(C)** shows the percentage of false alarm errors, i.e., uninhibited reactions to the non-target stimuli. All outcome measures are shown as means, which show the height of the bars, accompanied by the individual results of each participant indicated by points.

As for the performance in the n-back task at the three levels of difficulty, the results of the statistical analysis showed that the sensitivity index (*d*′), as an indicator of accuracy in the n-back task, depended significantly on both age [*F*_(1, 108)_ = 67.399; *p* < 0.001; η^2^*p* = 0.391] and the task difficulty level [*F*_(2, 216)_ = 204.370; *p* < 0.001; η^2^*p* = 0.661] but not the interaction between these two factors [*F*_(2, 216)_ = 2.854; *p* = 0.060; η^2^*p* = 0.026]. The *post-hoc* analysis showed that older and younger participants were significantly inferior (*p* < 0.001) in terms of accuracy in the n-back task at each difficulty level, even at the easiest one, i.e., 1-back. Moreover, performance accuracy decreased significantly (*p* < 0.001) in the successive levels of difficulty of the n-back task in both age groups. The pattern of declining accuracy as the level of difficulty of the n-back task increased appeared to be similar in both age groups (Figure 3A).

Analyses regarding correct responses to the target (%) revealed again significant differences related to age [*F*_(1, 108)_ = 11.062; *p* = 0.001; η^2^*p* = 0.095] and the difficulty of the task [*F*_(2, 216)_ = 103.735; *p* < 0.001; η^2^*p* = 0.497] but not to the interaction of these two factors [*F*_(2, 216)_ = 1.640; *p* = 0.194; η^2^*p* = 0.015]. According to the results of the *post-hoc* analysis, the percentage of correct answers on target decreased significantly according to the increasing difficulty of the task in both age groups, i.e., in older adults (1-back vs. 2-back and 2-back vs. 3-back: *p* < 0.001) and young adults (1-back vs. 2-back: *p* = 0.001 and 2-back vs. 3-back: *p* < 0.001). At the same time, differences driven by age in the correctness of responses to the target were found to be significant only for the 2-back level (*p* = 0.005) but not for the 1-back (*p* = 0.272) and 3-back (*p* = 0.540) levels (Figure 3B).

In contrast, for false alarm errors, age [*F*_(1, 108)_ = 66.288; *p* < 0.001; η^2^*p* = 0.387], level of task difficulty [*F*_(2, 216)_ = 85.539; *p* < 0.001; η^2^*p* = 0.449], and the interactive effect of these two variables [*F*_(2, 216)_ = 8.468; *p* < 0.001; η^2^*p* = 0.075] proved to be significant. The percentage of false alarm errors increased in both age groups with advancing task difficulty, with a tremendous increase in the older individuals (Figure 3C). As indicated by the *post-hoc* analysis, at all levels of task difficulty, older adults made significantly more false alarm errors than young adults (1-back: *p* = 0.006; 2-back and 3-back: *p* < 0.001). As for the rate of false alarm errors at successive levels of task difficulty, in the older adult group, the increase was significant at the *p* < 0.001 level between both 1-back and 2-back and 2-back and 3-back levels. In addition, the increase was significant in the young adult group between the 1-back and 2-back tasks (*p* = 0.034) and the 2-back and 3-back tasks (*p* = 0.002).

### Power of theta and alpha bands

#### Theta band

**Figure 4:**
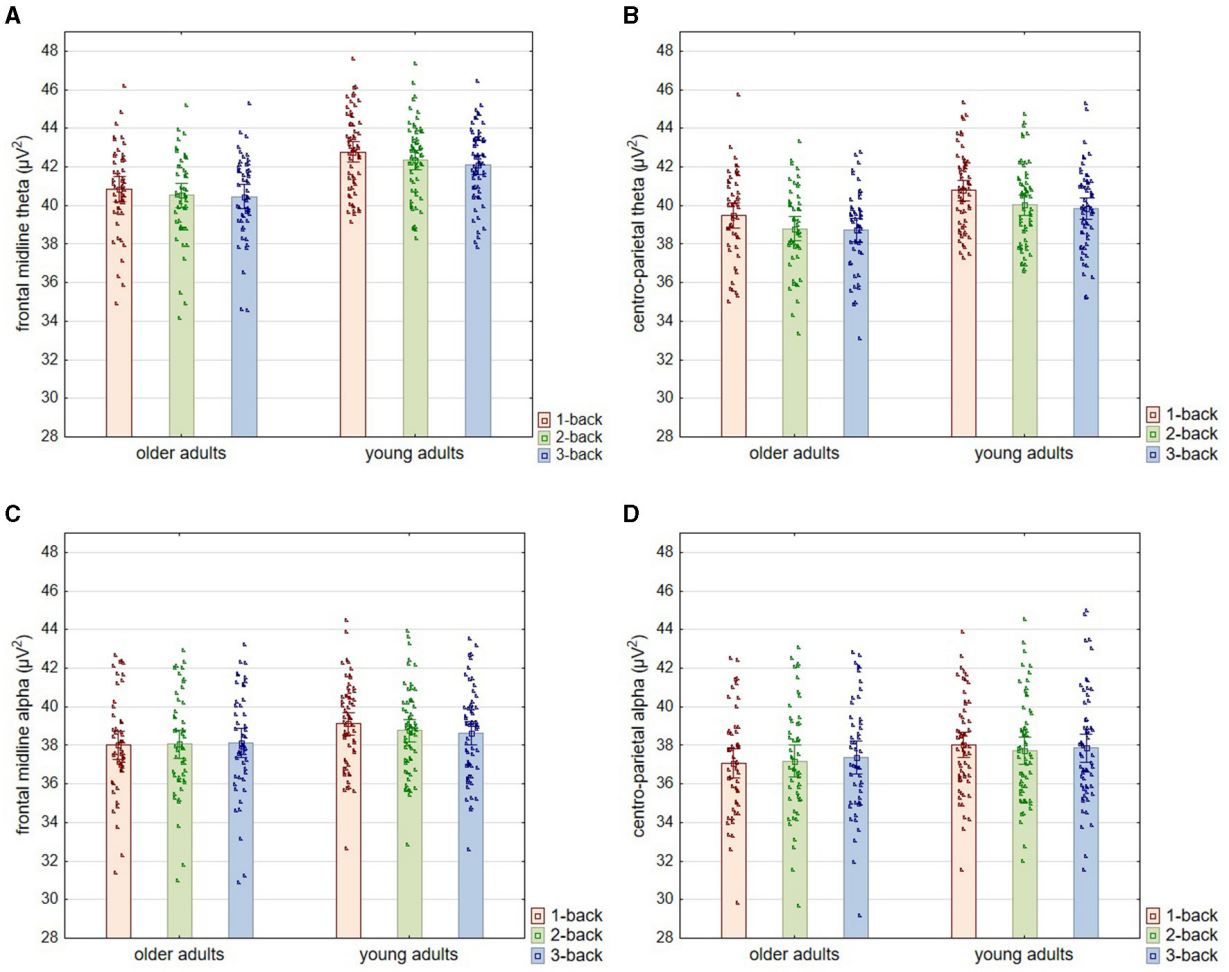
Theta and alpha power for three levels of n-back task difficulty in the older and young adult age groups. All plots provide the power (μV^2^) of a particular band according to the level of the task difficulty in two age groups: older and young adults. The top two plots **(A, B)** show the theta power, and the middle bottom two plots **(C, D)** show the alpha power results. The left two plots **(A, C)** show the power of a particular band (theta or alpha) measured from electrodes: Fz, AF3, AF4, F3, F4, FC1, and FC2 (frontal midline area). The right two plots **(B, D)** show the power of a particular band (theta or alpha) measured from electrodes: CP1, CP2, P3, P4, and Pz (centro-parietal area). All outcome measures are shown as means, which show the height of the bars, accompanied by the individual results of each participant indicated by points.

**Figure 5:**
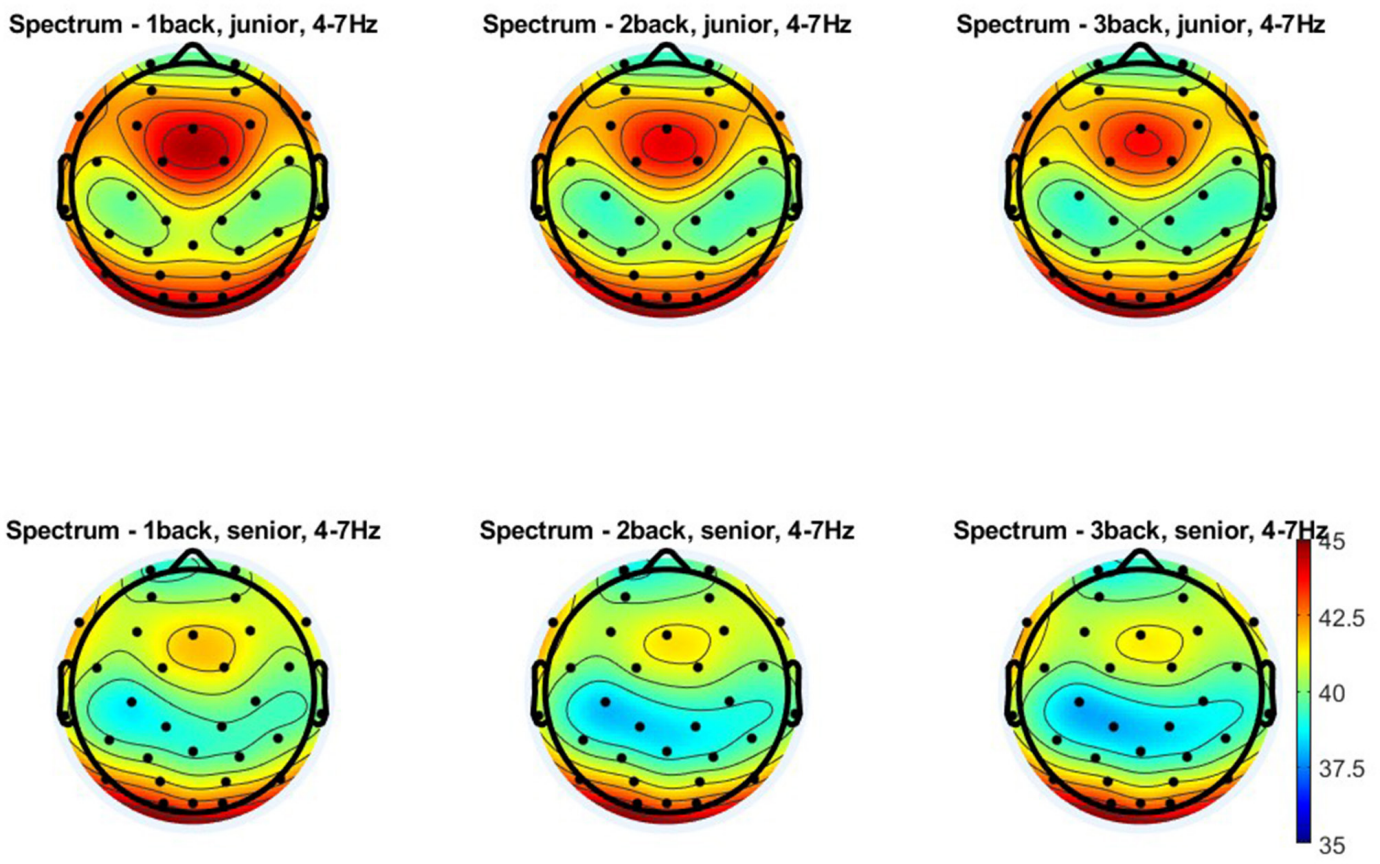
Topographical distribution of the theta power for three levels of n-back task difficulty in the older and young adult age groups. All plots provide the topographical distribution of the theta power (μV^2^) according to the level of the task difficulty in two age groups: older and young adults. The top three plots show the distribution of the theta power for 1-back, 2-back, and 3-back tasks obtained in young adults, labeled as “junior.” The bottom three plots show the distribution of the theta power for 1-back, 2-back, and 3-back tasks obtained in older adults, labeled as “senior.”

Regarding the theta oscillation measured with electrodes located in the frontal-midline region (as representing FMT), the analysis revealed significant differences in theta power according to age [*F*_(1, 108)_ = 21.160; *p* < 0.001; η^2^*p* = 0.164] and the level of the n-back task difficulty [*F*_(2, 216)_ = 27.540; *p* < 0.001; η^2^*p* = 0.203]. At the same time, the nature of the latter differences did not depend on the age of the participants—no interaction effect of age and task difficulty level was found [*F*_(2, 216)_ = 1.830; *p* = 0.163; η^2^*p* = 0.017]. *Post-hoc* analysis revealed that older adults have lower theta power than young adults at each level of difficulty of the n-back task, i.e., 1-back (*p* < 0.001), 2-back (*p* < 0.001), and 3-back (*p* = 0.002). In terms of differences relating to the task difficulty, theta power was significantly higher in the 1-back task than in the 2-back and 3-back tasks in both older (both *p* < 0.001) and young individuals (respectively *p* = 0.030 and *p* = 0.005). In contrast, there were no differences in theta power between the 2-back and 3-back conditions, again in both older (*p* = 0.167) and young (*p* = 0.993) adults (see Figures 4A, 5).

Similar results were obtained for theta measured from electrodes in the centro-parietal area, although the age differences observed here were more minor (Figures 4B, 5). Theta power differed significantly as a function of age [*F*_(1, 108)_ = 9.280; *p* = 0.003; η^2^*p* = 0.079] and the level of n-back task difficulty [*F*_(2, 216)_ = 67.590; *p* < 0.001; η^2^*p* = 0.385] but not as a function of the interaction of these two variables [*F*_(2, 216)_ = 0.730; *p* = 0.481; η^2^*p* = 0.007]. As the *post-hoc* analysis results indicated, significantly higher theta power in younger than older adults was observed only in the 1-back task (*p* = 0.033). At higher task difficulty levels, age-related differences were not significant (2-back: *p* = 0.053 and 3-back: *p* = 0.104). Differences in theta power measured in the centro-parietal region appeared to follow a pattern parallel to that observed for FMT. Theta power was again significantly higher in the 1-back conditions in comparison to 2-back and 3-back conditions in both age groups, older adults (*p* < 0.001) and young adults (*p* < 0.001). At the same time, there were no differences in theta power between 2-back and 3-back tasks in older adults (*p* = 0.526) and young adults (*p* = 0.994).

#### Alpha band

**Figure 6:**
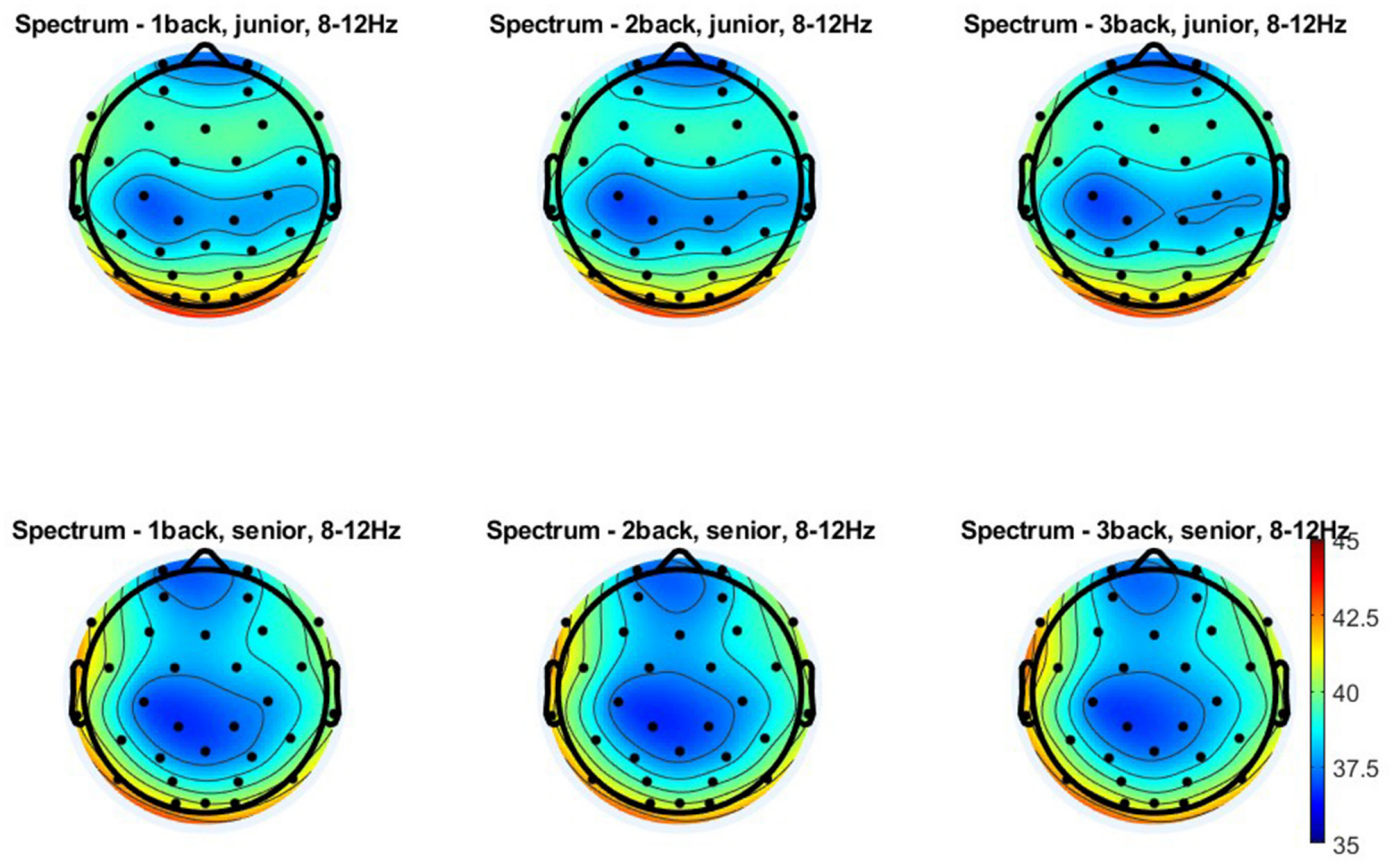
Topographical distribution of the alpha power for three levels of n-back task difficulty in the older and young adult age groups. All plots provide the topographical distribution of the alpha power (μV^2^) according to the level of the task difficulty in two age groups: older and young adults. The top three plots show the distribution of the alpha power for 1-back, 2-back, and 3-back tasks obtained in young adults, labeled as “junior.” The bottom three plots show the distribution of the alpha power for 1-back, 2-back, and 3-back tasks obtained in older adults, labeled as “senior.”

As regards the alpha power measured in the frontal-midline area (Figures 4C, 6), the analysis indicated no differences related to age [*F*_(1, 108)_ = 2.500; *p* = 0.092; η^2^*p* = 0.026] or the level of task difficulty [*F*_(2, 216)_ = 0.730; *p* = 0.084; η^2^*p* = 0.023]. However, the interaction effect of age and the task difficulty level proved significant [*F*_(2, 216)_ = 5.710; *p* = 0.004; η^2^*p* = 0.050]. According to the *post-hoc* analysis, there were no age-related differences in alpha power at any task difficulty levels (1-back: *p* = 0.222; 2-back: *p* = 0.697; 3-back: *p* = 0.893). In the group of young adults, the alpha power accompanying the performance of the easiest task (1-back) was significantly higher than that of the two more difficult versions (2-back: *p* = 0.003 and 3-back: *p* = 0.001). In comparison, there were no differences between these two last versions (*p* = 0.931). In contrast, the group of older adults showed no differences in alpha power between n-back tasks of varying difficulty (1-back vs. 2-back: *p* = 1.00; 1-back vs. 3-back: *p* = 0.956; 2-back vs. 3-back: *p* = 0.993).

The analysis of alpha power measured in the centro-parietal area provided similar results (Figures 4D, 6). It revealed a lack of differences in alpha power related to age [*F*_(1, 108)_ = 1.620; *p* = 0.206; η^2^*p* = 0.015] and the level of task difficulty [*F*_(2, 216)_ = 1.250; *p* = 0.289; η^2^*p* = 0.011], with a significant interaction effect of these two factors [*F*_(2, 216)_ = 3.790; *p* = 0.024; η^2^*p* = 0.034]. Based on the *post-hoc* analysis results, there were no differences between age groups for each level of task difficulty (1-back: *p* = 0.513; 2-back: *p* = 0.921; 3-back: *p* = 0.957). Moreover, despite the significant interaction effect, there was also no significant difference in alpha power depending on the level of difficulty of the n-back task in both studied age groups: older adults (1-back vs. 2-back: *p* = 0.952; 1-back vs. 3-back: *p* = 0.232; 2-back vs. 3-back: *p* = 0.765) and young adults (1-back vs. 2-back: *p* = 0.217; 1-back vs. 3-back: *p* = 0.745; 2-back vs. 3-back: *p* = 0.953).

## Discussion

The theoretical context of the current study was the phenomenon of compensatory brain activity in older adults and, more specifically, the CRUNCH hypothesis. We examined the performance on the n-back task and the power of theta and alpha oscillations according to the task's difficulty level and the participant's age in the context of the CRUNCH hypothesis.

Our assumptions regarding the cognitive performance of the n-back task were only partially confirmed. Working memory proved to be weaker in older adults than in young adults. However, age-based differences in two indicators, namely the sensitivity index (*d*′) and the percentage of false alarm errors, were apparent even at the lowest level of the task difficulty, i.e., 1-back, which was not the expected result (see Bopp and Verhaeghen, 2020). This finding would mean that, even with a low load on WM in terms of updating the stored information, older adults are not able to function at the level of young individuals. Regarding correct answers to the target, older and younger participants differed only in the intermediate difficulty level of the task–2-back. The lack of age-related differences for the easiest task (1-back) can be interpreted as resulting from older adults' sufficient neurocognitive resources to function at the level of young persons. In contrast, the lack of age-related differences in the most difficult task (3-back) results from such demands of the task for which the resources of young individuals are also insufficient.

What is essential to note from the perspective of the aim of the current study is that the optimal situation for testing the CRUNCH hypothesis and its predictions could not be fully reconstructed. For the lowest difficulty level of the task, by assuming it to be the least neurocognitively demanding, the performance of the older adults was just as good as that of the young ones in terms of the percentage of correct responses to the target, but it was also worse in terms of false alarm errors and overall performance accuracy indicated by sensitivity index (*d*′). At the same time, it should be noted that the performance of the 1-back task by the older participants cannot be considered poor (on a mean, they responded correctly to 85% of the target stimuli, made false alarm errors in response to 10% of non-targets and the mean *d*′ in this age group was 2.7). Therefore, even though the performance of the older individuals was not at the level of the younger ones, they remained effective in the 1-back task. Hence, it can still be assumed that the 1-back task created conditions in which compensatory brain activity in older participants could be engaged, although not enough to keep their performance at the level of young adults.

Consequently, the hypotheses regarding the compensatory brain activity of older adults concerning both theta and alpha power have not been confirmed.

Older participants showed lower frontal midline theta power than young adults, regardless of the level of cognitive demands of the task, which was accompanied by poorer task performance in this age group. This result is consistent with those obtained in other studies (Belham et al., 2013; Gajewski and Falkenstein, 2014) and can be interpreted as the limited ability of older adults to engage frontal resources responsible for executive control, including WM. There are also reports of results to the contrary, i.e., an increase in frontal and temporal theta power in older adults (Huizeling et al., 2021), which the authors interpret, taking caution, as a manifestation of compensatory brain activity. However, the results obtained in the current study are instead in line with the assumption that a decline in theta power can be considered a marker of neurocognitive aging in WM (Cummins and Finnigan, 2007; van de Vijver et al., 2014; Reichert et al., 2016). We also observed lower theta power in older than young participants in the centro-parietal area. However, in this case, age-related differences are more minor and are statistically significant only for the easiest level of the n-back task. Considering the generally lower theta power measured from the centro-parietal area compared to the frontal midline, it can be concluded that theta oscillations in the centro-parietal area are less critical for the performance of the n-back task than FMT.

Expanding on the previous point, the changes in theta power observed in this study with a rise in the level of the task difficulty are contrary to the most commonly observed increase in theta with growing cognitive load (e.g., Gevins et al., 1997; Constantinidis and Klingberg, 2016). We observed a significant decrease in theta power measured in the frontal midline and centro-parietal areas with increasing cognitive demands. Such a pattern in older adults could be interpreted as compensatory only if it was the opposite in young adults and if, in the older participants, a low cognitive load would be accompanied by theta of a higher power than in young adults, as in the study by McEvoy et al.'s (2001), for example. However, in our study, there was a decrease in theta power with increasing cognitive demand in both age groups. Thus, regardless of age, it would instead reflect the deteriorating performance observed at subsequent difficulty levels of the n-back task. The relationship between poorer cognitive performance and lower theta power has been previously reported in research (e.g., Belham et al., 2013; Gajewski and Falkenstein, 2014) and interpreted as lower neurocognitive resources resulting in weaker cognitive functioning.

When it comes to alpha power, the age of the participants did not differentiate it, regardless of the level of cognitive load and the area in which alpha power was registered. In general, alpha power also did not show changes analogous to those observed for theta power with increasing cognitive demands. Only in the young adult group was alpha power measured in the frontal midline area higher at the lowest level of cognitive load than in the two higher levels. Since a decrease in alpha power in an area associated with a specific cognitive activity corresponds to involvement in that activity, which in the case of WM information updating (n-back task) concerns the frontal, central, and parietal areas (Tsoneva et al., 2011), a decrease in alpha power in the midline frontal area when moving from a 1-back to a 2-back task can be interpreted as an adaptation to increasing cognitive demands of the task. The behavioral results are consistent with this finding, showing that the level of performance on the 1-back task by young participants is very high, indicating the high ease of this task in this age group. In this context, this kind of neural adaptation to the increasing difficulty of the task does not appear in older adults. This finding is unlike that obtained in another study, which was also conducted among older adults using the n-back task (Gajewski and Falkenstein, 2014). However, that study compared the alpha power accompanying the performance of 0-back and 2-back tasks. This result implies a lower cognitive demand for the easier task compared to the current study and, second, a greater difference in the difficulty level of the tasks for which alpha power was compared. These differences in the characteristics of both studies may explain the different findings.

Moreover, the results indicate that the theta band is more related to involvement in n-back tasks requiring information updating in WM than the alpha band, which is consistent with previous knowledge.

In summary, the analysis of the theta and alpha wave power accompanying the performance of tasks involving WM at different difficulty levels did not confirm the hypotheses regarding compensatory brain activity in older adults. The results showed a reduced capacity to use neuronal resources relevant to this task in older adults rather than compensatory activity. Lower theta power in the older individuals was observed even at the lowest level of task difficulty (1-back), where compensatory activity was expected. Although already at this level of difficulty, cognitive performance was worse in older adults than in young adults, which could reduce the chances of observing the compensatory brain activity; in general, the cognitive performance of the 1-back task in the older group can be considered good, which, in turn, would support the presence of the compensatory brain activity. In addition, and importantly, the changes in theta power observed in both age groups accompanying growing task difficulty reflected a declining capacity for efficient cognitive functioning under increasing demands rather than an adaptation to this increase. As for the alpha band, on the other hand, in young adults, the decrease in its power to some extent reflected adaptation to increasing cognitive demands, but in older adults, no analogous pattern was observed.

The current study was not free of limitations. First and foremost, the introduced n-back task difficulty levels did not allow optimal inference of compensatory brain activity since even at the lowest difficulty level, older adults performed worse than young adults in light of two out of three indicators considered. In addition, the samples of older and young adults were not of equal size. They were also not gender balanced. Moreover, the age groups of older and young adults differed significantly in terms of educational level and number of years of education. However, such differences in educational attainment between generations are typical of Polish society. Finally, the older adults' raw scores on fluid intelligence (Raven's test) were significantly lower than those of the young participants, which, however, is also in line with knowledge of changes in fluid intelligence with age.

As regards directions for further research, it is undoubtedly worthwhile to ensure that the task difficulty levels are selected in such a way as to reproduce the conditions described in the predictions of the CRUNCH hypothesis. It would also be helpful to measure theta and alpha for a larger number of diverse cognitive functions, including those that involve executive control to a lesser extent than the n-back task (involving WM, which is categorized as an executive function). It should also be interesting to check neuroimaging (e.g., fMRI) and electrophysiological (EEG) indicators of compensatory brain activity from the same participants in a single study. It is also possible to expand the research and data analysis to include brain wave frequencies and regions other than those chosen in the current study.

## Data availability statement

The raw data supporting the conclusions of this article will be made available by the authors, without undue reservation.

## Ethics statement

The studies involving humans were approved by Bioethics Committee of the Nicolaus Copernicus University in Toruń functioning at Collegium Medicum in Bydgoszcz. The studies were conducted in accordance with the local legislation and institutional requirements. The participants provided their written informed consent to participate in this study.

## Author contributions

LZ-L: Conceptualization, Data curation, Formal analysis, Funding acquisition, Investigation, Methodology, Project administration, Resources, Supervision, Validation, Visualization, Writing – original draft, Writing – review & editing. EZ-M: Data curation, Formal analysis, Methodology, Writing – original draft, Writing – review & editing. DZ: Data curation, Formal analysis, Methodology, Writing – original draft, Writing – review & editing. PA: Data curation, Formal analysis, Software, Writing – original draft, Writing – review & editing.
