# Supplementary material for: Compensatory brain activity pattern is not present in older adults during the n-back task performance—Findings based on EEG frequency analysis
Source: Front Psychol. 2024 Apr 11;15:1371035. doi: 10.3389/fpsyg.2024.1371035 (PMC11043891; doi:10.3389/fpsyg.2024.1371035)
Supplement: Supplementary file 1 [file Table_1.DOC]

Supplementary Material

**Table S1**

*Comparison of older and young adults in terms of reaction times in the n-back task at three levels of difficulty*

|  | Older adults | Young adults | Student’s *t* | *p*-value | Welch’s *t* | *p*-value | Hotelling *T2* |
| --- | --- | --- | --- | --- | --- | --- | --- |
|  | *M (SD)* | *M (SD)* |  |  |  |  |  |
| 1-back | 0,774 (0.146) | 0.585 (0.107) | 7.702 | < .001 | 7.488 | < .001 | *T2* = 57.370  *p* < .001 |
| 2-back | 0.884 (0.194) | 0.740 (0.174) | 4.090 | < .001 | 4.047 | < .001 |  |
| 3-back | 0.841 (0.159) | 0.690 (0.132) | 5.420 | < .001 | 5.329 | < .001 |  |

*Note.* Response time is expressed in milliseconds

**Table S2**

*Correlation between reaction time and the indicators of the accuracy in the n-back task at three levels of difficulty in older and young adult age groups*

|  |  | Older adults | | Young adults | |
| --- | --- | --- | --- | --- | --- |
|  |  | Pearson’s *r* | *p*-value | Pearson’s *r* | *p*-value |
|  |  | RT in 1-back task | | RT in 1-back task | |
| 1-back | d’ | 0.143 | .324 | 0.161 | .228 |
|  | Corr % | - 0.154 | .289 | 0.075 | .574 |
|  | FA % | - 0.188 | .195 | - 0.018 | .892 |
|  |  | RT in 2-back task | | RT in 2-back task | |
| 2-back | d’ | - 0.093 | .524 | - 0.199 | .135 |
|  | Corr % | - 0.222 | .125 | - 0.060 | .653 |
|  | FA % | - 0.241 | .095 | 0.211 | .112 |
|  |  | RT in 3-back task | | RT in 3-back task | |
| 3-back | d’ | 0.078 | .594 | - 0.026 | .848 |
|  | Corr % | 0.024 | .868 | 0.153 | .251 |
|  | FA % | - 0.033 | .822 | 0.185 | .164 |

*Note.* Particular abbreviations included in the table denote: d’ – sensitivity index; Corr % – the percentage of the correct responses to the target; FA % – the percentage of the false alarm errors; RT – mean reaction time (in milliseconds)
